# The Natural Flavone Acacetin Blocks Small Conductance Ca^2+^-Activated K^+^ Channels Stably Expressed in HEK 293 Cells

**DOI:** 10.3389/fphar.2017.00716

**Published:** 2017-10-10

**Authors:** Kui-Hao Chen, Hui Liu, Hai-Ying Sun, Man-Wen Jin, Guo-Sheng Xiao, Yan Wang, Gui-Rong Li

**Affiliations:** ^1^Department of Medicine, Li Ka Shing Faculty of Medicine, University of Hong Kong, Hong Kong, Hong Kong; ^2^Department of Pharmacology, Tongji Medical College, Huazhong University of Science and Technology, Wuhan, China; ^3^Xiamen Cardiovascular Hospital, Xiamen University, Xiamen, China

**Keywords:** acacetin, ion channels, potassium channels, small conductance Ca^2+^-activated potassium channels

## Abstract

The natural flavone acacetin inhibits several voltage-gated potassium currents in atrial myocytes, and has anti-atrial fibrillation (AF) effect in experimental AF models. The present study investigates whether acacetin inhibits the Ca^2+^-activated potassium (K_Ca_) currents, including small conductance (SK_Ca_1, SK_Ca_2, and SK_Ca_3), intermediate conductance (IK_Ca_), and large-conductance (BK_Ca_) channels stably expressed in HEK 293 cells. The effects of acacetin on these K_Ca_ channels were determined with a whole-cell patch voltage-clamp technique. The results showed that acacetin inhibited the three subtype SK_Ca_ channel currents in concentration-dependent manner with IC_50_ of 12.4 μM for SK_Ca_1, 10.8 μM for SK_Ca_2, and 11.6 μM for SK_Ca_3. Site-directed mutagenesis of SK_Ca_3 channels generated the mutants H490N, S512T, H521N, and A537V. Acacetin inhibited the mutants with IC_50_ of 118.5 μM for H490N, 275.2 μM for S512T, 15.3 μM for H521N, and 10.6 μM for A537V, suggesting that acacetin interacts with the P-loop helix of SK_Ca_3 channel. However, acacetin at 3–10 μM did not decrease, but induced a slight increase of BK_Ca_ (+70 mV) by 8% at 30 μM. These results demonstrate the novel information that acacetin remarkably inhibits SK_Ca_ channels, but not IK_Ca_ or BK_Ca_ channels, which suggests that blockade of SK_Ca_ by acacetin likely contributes to its anti-AF property previously observed in experimental AF.

## Introduction

Potassium channels are the largest and the most diverse super-family of ion channels in living organisms from bacteria and insects to animals including humans. Among them, Ca^2+^-activated potassium channels (K_Ca_) comprise many members. They are divided into three subfamilies: big (or large) conductance (BK_Ca_, Slo, or K_Ca_1.1, encoded by *KCNMA1*), intermediate conductance (IK_Ca_ or K_Ca_3.1, encoded by *KCNN4*), and small conductance channels (SK_Ca_1, SK_Ca_2, and SK_Ca_3 or K_Ca_2.1, K_Ca_2.2, and K_Ca_2.3, encoded by *KCNN1, KCNN2*, and *KCNN3*, respectively) (Girault et al., 2012; Gueguinou et al., 2014). The three SK_Ca_ channels are expressed in excitable tissues (e.g., neurons, skeletal muscle, adrenal gland, and heart) and also in some non-excitable tissues (e.g., liver, vascular endothelium, cancers, etc.) (Wei et al., 2005). In neurons, apamin-sensitive SK_Ca_ current is responsible for afterhyperpolarization (Weatherall et al., 2010) and regulates firing frequency as well as learning and memory (Adelman et al., 2012). In the cardiovascular system, SK_Ca_ channels contribute to cardiac repolarization (Xu et al., 2003; Li et al., 2009; Zhang et al., 2014), endothelium-derived hyperpolarization-type arterial dilation in response to increased hemodynamics (Wulff and Kohler, 2013), and also provide negative feedback on sympathetic tone (Taylor et al., 2003). Results from recent studies suggest that SK_Ca_ channels play a role in atrial fibrillation (AF) (Diness et al., 2010; Ellinor et al., 2010; Qi et al., 2014; Haugaard et al., 2015), tumor cell migration and metastasis (Chantome et al., 2013), and overactive bladder (Soder et al., 2013). A recent report demonstrated that the SK_Ca_ inhibitor apamin may cause ventricular arrhythmias in failing rabbit hearts (Chang et al., 2013); however, blockade of SK_Ca_ channels is very effective in anti-AF (Diness et al., 2010; Qi et al., 2014; Haugaard et al., 2015). The development of SK channel blockers has been considered as a new therapeutic strategy in the treatment of AF (Zhang et al., 2015).

We have previously reported that the natural flavone acacetin from the traditional Chinese medicinal herb Xuelianhua (*Saussurea involucrata*) prolongs the atrial effective refractory period and prevents or terminates the experimentally induced AF in anesthetized dogs without increasing the QT interval (Li et al., 2008; Liu et al., 2016) by inhibiting atrial *I*_Kur_ (ultra-rapidly activating delayed rectifier potassium current) or Kv1.5, *I*_K.ACh_ (acetylcholine-activated potassium current), and *I*_to_ (transient outward potassium current) (Wu et al., 2011, 2013a). The present study investigated the effects of acacetin on SK_Ca_1, SK_Ca_2, SK_Ca_3, IK_Ca_, and BK_Ca_ currents in HEK 293 cells stably expressing corresponding channel genes with a conventional whole-cell patch voltage-clamp technique. Our results showed that acacetin inhibited the three subtypes of SK_Ca_ channels, but not IK_Ca_ and BK_Ca_ channels, suggesting that the blockade of SK_Ca_ channels may also participate in the anti-AF previously observed in experimental canine models.

## Materials and Methods

### Cell Line Culture and Gene Transfection

The pCDNA3/rSK_Ca_2 (*KCNN2*), pCDNA3/hSK_Ca_3 (*KCNN3*), and pCDNA3/hIK_Ca_ (*KCNN4*) plasmids obtained as generous gifts from Dr. Nicole Schmitt (Department of Biomedical Sciences, University of Copenhagen, Copenhagen, Denmark) were transfected into HEK 293 cells (ATCC, Manassas, VA, United States) using Lipofectamine 2000^TM^. The HEK 293 cell lines stably expressing the SK_Ca_1, SK_Ca_2, and SK_Ca_3 channels were established as described previously (Wu et al., 2012). The cell lines were maintained in Dulbecco’s modified Eagle’s medium (Invitrogen, Hong Kong, China) supplemented with 10% fetal bovine serum and G418 (400 μg/ml). HEK 293 cell line (Wu et al., 2013c) stably expressing human BK_Ca_ (*KCNMA1*) was also maintained in the same culture conditions. Cells were seeded on glass cover slips for electrophysiological recording.

The primers of SK_Ca_3 mutants were synthesized by the Genome Research Center, the University of Hong Kong (Hong Kong), and the mutants were generated using a QuikChange kit (Stratagene, La Jolla, CA, United States). After confirmed by DNA sequencing, the mutants were transiently expressed in HEK 293 cells in a 35 mm culture dish using Lipofectamine 2000^TM^ (10 μl) with SK_Ca_3 mutant cDNA plasmid (4 μg).

### Drugs and Solutions

Acacetin (5,7-dihydroxy-4′-methoxyflavone) was synthesized in the laboratory as described previously in the US patent (Li et al., 2010). The stock solution (100 mM) of acacetin was prepared with dimethyl sulfoxide, aliquoted, and stored at -20°C. Tyrode’s solution used in this study contained (in mM): 140 NaCl, 5.4 KCl, 1 MgCl_2_, 1.8 CaCl_2_, 10 HEPES, and 10 glucose (pH was adjusted to 7.3 with NaOH). The pipette solution contained (in mM): 20 KCl, 110 potassium aspartate, 1.0 MgCl_2_, 10 HEPES, 5 EGTA, 0.1 GTP, 5 sodium phosphocreatine, and 5 Mg-ATP, pH adjusted to 7.2 with KOH (Wu et al., 2013b), in which 300 nM free Ca^2+^ (calculated using the Cabuf software provided by Dr. G. Droogmans, Department of Physiology, KU Leuven, Leuven, Belgium) was included.

### Electrophysiology

The HEK 293 cells on a coverslip were placed into a cell chamber mounted on the stage of an inverted microscope (Olympus, IX70, Japan), and superfused with Tyrode’s solution (2 ml/min). Whole-cell current was recorded with a patch clamp amplifier (EPC-10, HEKA Elektronik, Lambrecht, Germany) as described previously (Wu et al., 2011, 2012, 2013c; Sun et al., 2014). Briefly, glass electrodes (1.2 mm OD) were pulled with a Brown–Flaming puller (Model P-97, Sutter Instrument Co., Novato, CA, United States). Resistance of the glass pipettes was 2–3 MΩ when filled with the pipette solution. Whole-cell configuration was established by a gentle suction after a gigaohm-seal was obtained. Electrical signal was stored on the hard disk of a PC computer. All experiments were performed at room temperature (22–23°C).

### Statistical Analysis

The data were expressed as means ± SEM. Unpaired Student’s *t*-tests were used as appropriate to evaluate the differences between two group means, and ANOVA was used for multiple groups. A value of *P* < 0.05 was considered to indicate statistical significance.

## Results

### Effect of Acacetin on SK_Ca_1 Current

**FIGURE 1:**
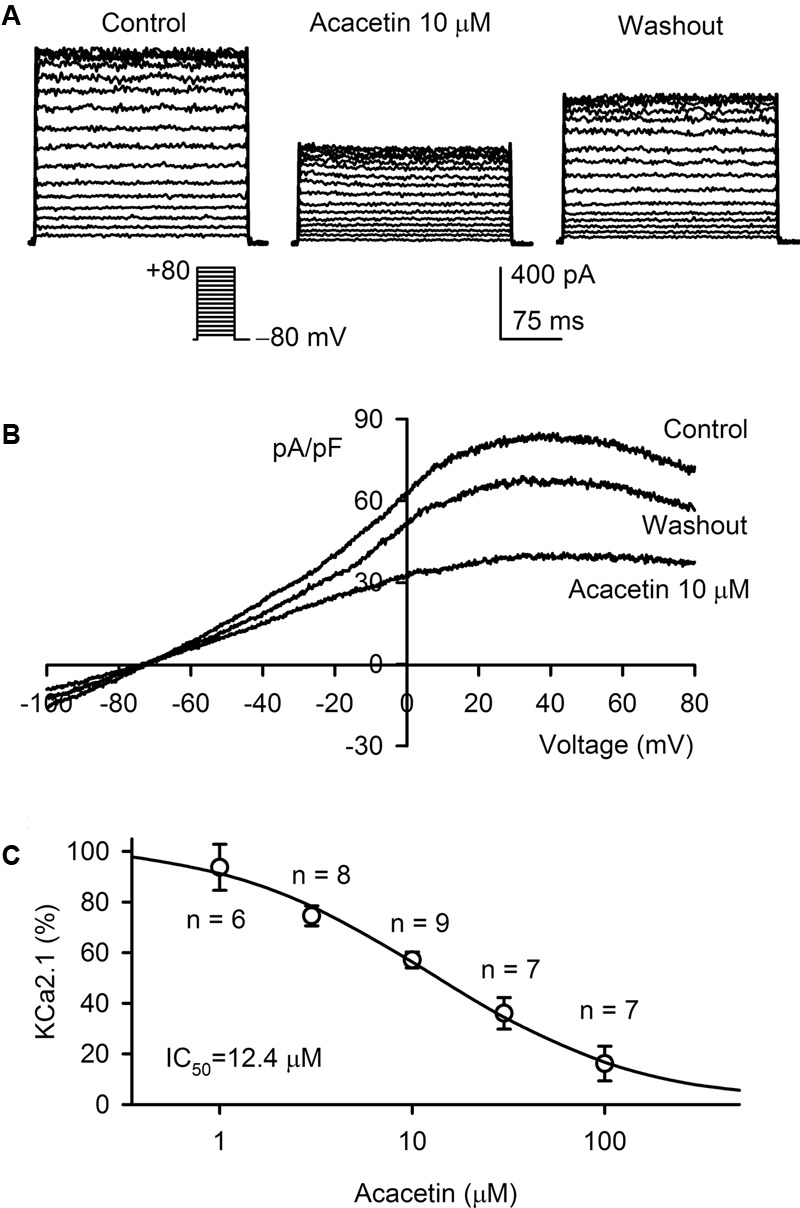
Effect of acacetin on SK_Ca_1 channel stably expressed in HEK 293 cells. **(A)** SK_Ca_1 current was activated in a representative cell expressing human *KCNN1* by 200-ms step voltages between –70 and +80 mV from a holding potential of –80 mV in the absence and presence of 10 μM acacetin. **(B)** Current–voltage (*I–V*) relationships of SK_Ca_1 current were recorded in a typical experiment with a 3-s voltage ramp from –100 to +80 mV in the absence and presence of 10 μM acacetin. **(C)** Concentration–response relationship of acacetin for inhibiting SK_Ca_1 current (+80 mV) was fitted to a Hill equation to obtain IC_50_ value of acacetin.

The effect of acacetin on SK_Ca_1 current was determined in HEK 293 cells stably expressing human *KCNN1*. **Figure 1A** displays the voltage-dependent SK_Ca_1 current recorded with 200-ms voltage steps between -70 and +80 mV from a holding potential of -80 mV in a representative cell. The current was inhibited by 10 μM acacetin (10 min exposure), and the inhibition was partially reversed by washout. **Figure 1B** displays the current–voltage (*I–V*) relationships of SK_Ca_1 determined in another typical experiment with a voltage ramp in the absence and presence of acacetin. The *I–V* relationships of SK_Ca_1 current showed a reversal potential around -70 mV and an inward rectification property, typical SK_Ca_ current as described previously (Girault et al., 2011; Wu et al., 2013b). The current was significantly descreased by 10 μM acacetin in bath solution, and the inhibition was partially reversed on washout. **Figure 1C** illustrates the concentration-dependent inhibition of SK_Ca_1 current (at +80 mV) by acacetin. The concentration–response curve was fitted to a Hill equation to obtain IC_50_ (the concentration of inhibiting 50% current) value. The IC_50_ of acacetin for inhibiting SK_Ca_1 at +80 mV was 12.4 μM (Hill co-efficient, 0.8).

### Effect of Acacetin on SK_Ca_2 Current

**FIGURE 2:**
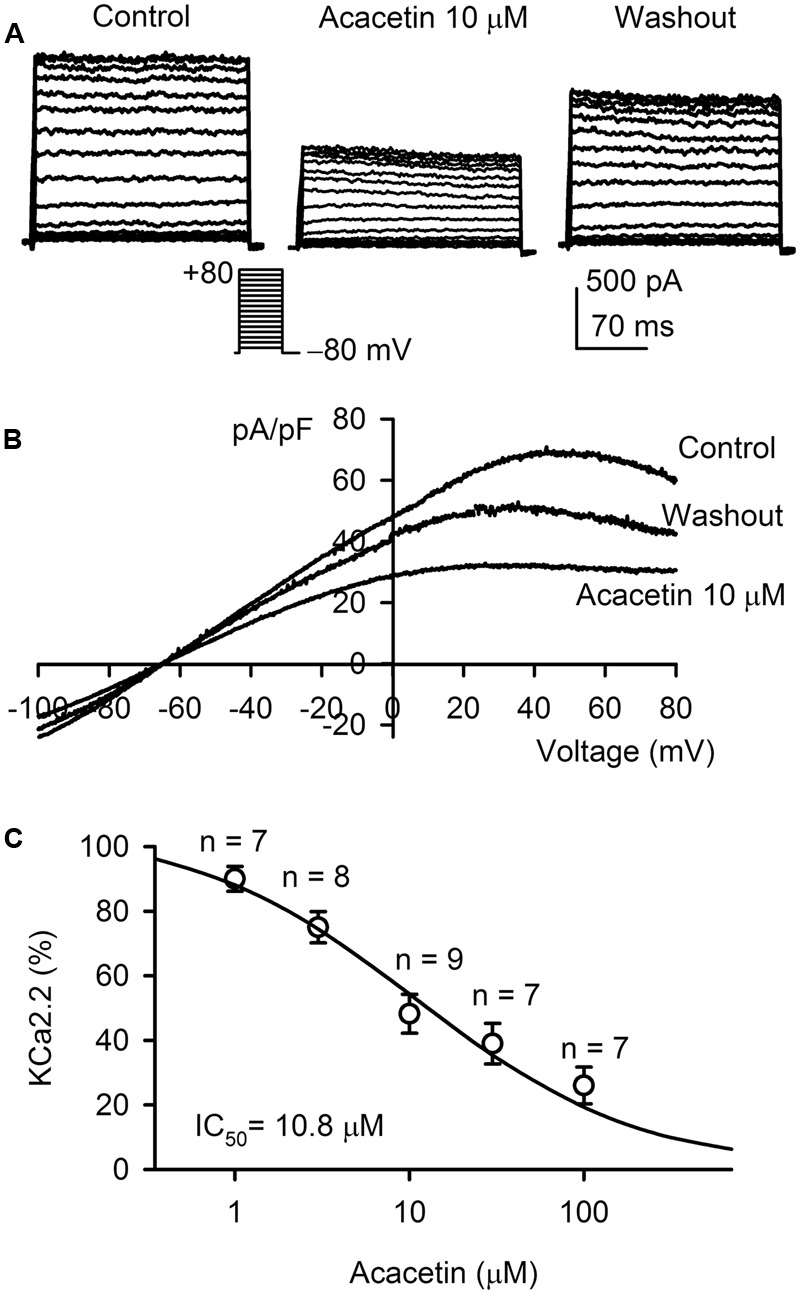
Effect of acacetin on SK_Ca_2 channel stably expressed in HEK 293 cells. **(A)** SK_Ca_2 current was activated in a representative cell expressing rat *KCNN2* by 200-ms step voltages between –70 and +80 mV from a holding potential of –80 mV in the absence and presence of 10 μM acacetin. **(B)** Current–voltage (*I–V*) relationships of SK_Ca_2 current were recorded in a typical experiment with a 3-s voltage ramp from –100 to +80 mV in the absence and presence of 10 μM acacetin. **(C)** Concentration–response relationship of acacetin for inhibiting SK_Ca_2 current (+80 mV) was fitted to a Hill equation to obtain IC_50_ value of acacetin.

The effect of acacetin on SK_Ca_2 current was determined in HEK 293 cell line expressing rat *KCNN2*. **Figure 2A** shows the voltage-dependent SK_Ca_2 current in a typical experiment with the voltage protocol as shown in the inset. The current was significantly decreased by 10 μM acacetin (10 min exposure) at all testing potentials, and the inhibition was partially reversed by washout. *I–V* relationships of SK_Ca_2 current determined by a ramp voltage protocol also showed an inward rectification. Inward and outward components of the current were decreased by 10 μM acacetin, and the inhibition was partially recovered on drug washout (**Figure 2B**). **Figure 2C** illustrates the concentration–response relationship of acacetin for inhibiting SK_Ca_2 current (at +80 mV). The curve was fitted to a Hill equation. The IC_50_ of acacetin for inhibiting SK_Ca_2 current was 10.8 μM (Hill coefficient, 0.8).

### Inhibition of SK_Ca_3 Current by Acacetin

**FIGURE 3:**
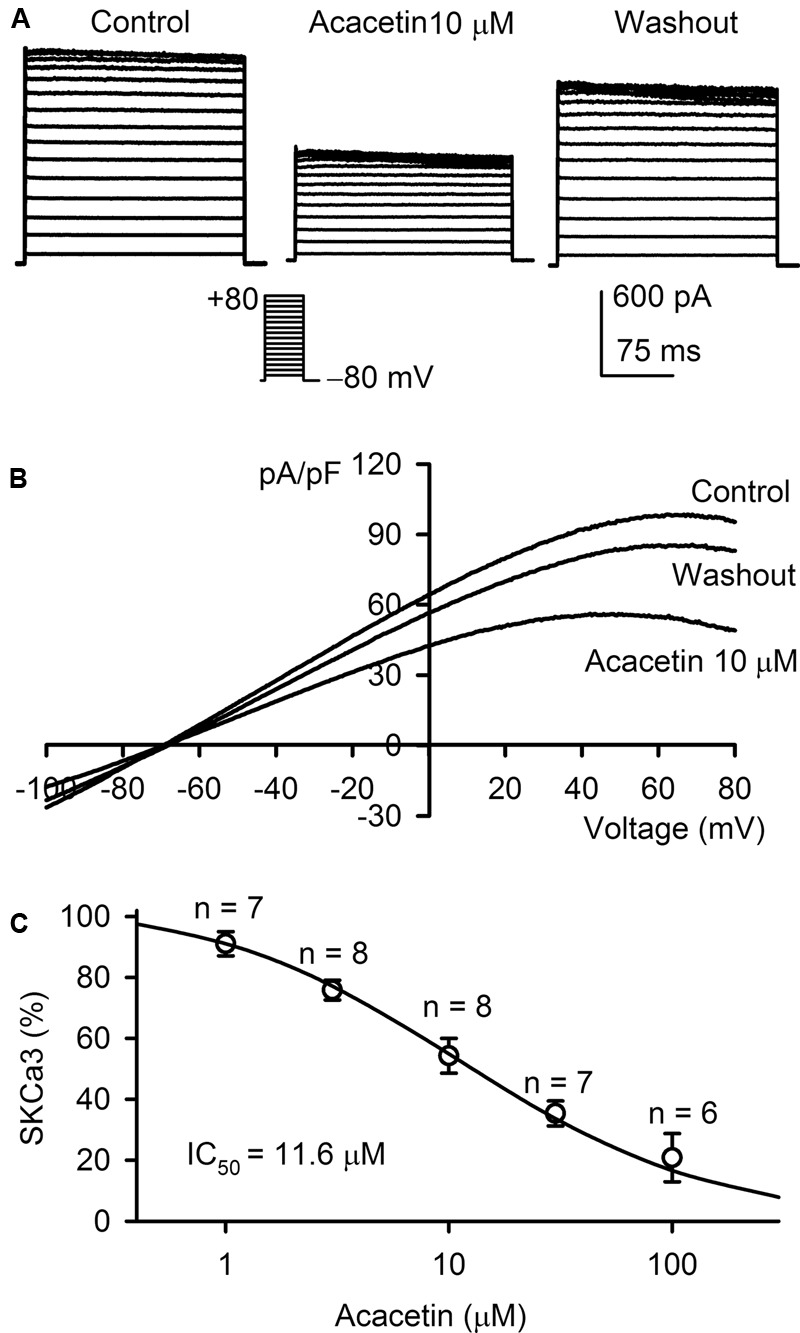
Effect of acacetin on SK_Ca_3 channel stably expressed in HEK 293 cells. **(A)** SK_Ca_3 current was activated in a representative cell expressing human *KCNN3* by 200-ms step voltages between –70 and +80 mV from a holding potential of –80 mV in the absence and presence of 10 μM acacetin. **(B)** Current–voltage (*I–V*) relationships of SK_Ca_3 current were recorded in a typical experiment with a 3-s voltage ramp from –100 to +80 mV in the absence and presence of 10 μM acacetin. **(C)** Concentration–response relationship of acacetin for inhibiting SK_Ca_3 current (+80 mV) was fitted to a Hill equation to obtain IC_50_ value of acacetin.

The inhibitory effect of acacetin on SK_Ca_3 was determined in HEK 293 cell line expressing human *KCNN3* gene. The voltage-dependent step SK_Ca_3 current was determined with the voltage protocol as shown in the inset in a typical experiment (**Figure 3A**). SK_Ca_3 current at all test potentials was inhibited by 10 μM acacetin with 10 min incubation, and the inhibition partially recovered on washout for 10 min. *I–V* relationships of SK_Ca_3 current was determined with a ramp voltage protocol in another cell (**Figure 3B**) before and after application of acacetin. The current also displays an inward rectification and was reversibly decreased by 10 μM acacetin. The concentration–response curve of acacetin for inhibiting SK_Ca_ 3 current was fitted to a Hill equation (**Figure 3C**). The IC_50_ of acacetin for inhibiting SK_Ca_3 was 11.6 μM (with a Hill coefficient of 0.8).

### Molecular Determinant of Acacetin for Inhibiting SK_Ca_ Channels

**FIGURE 4:**
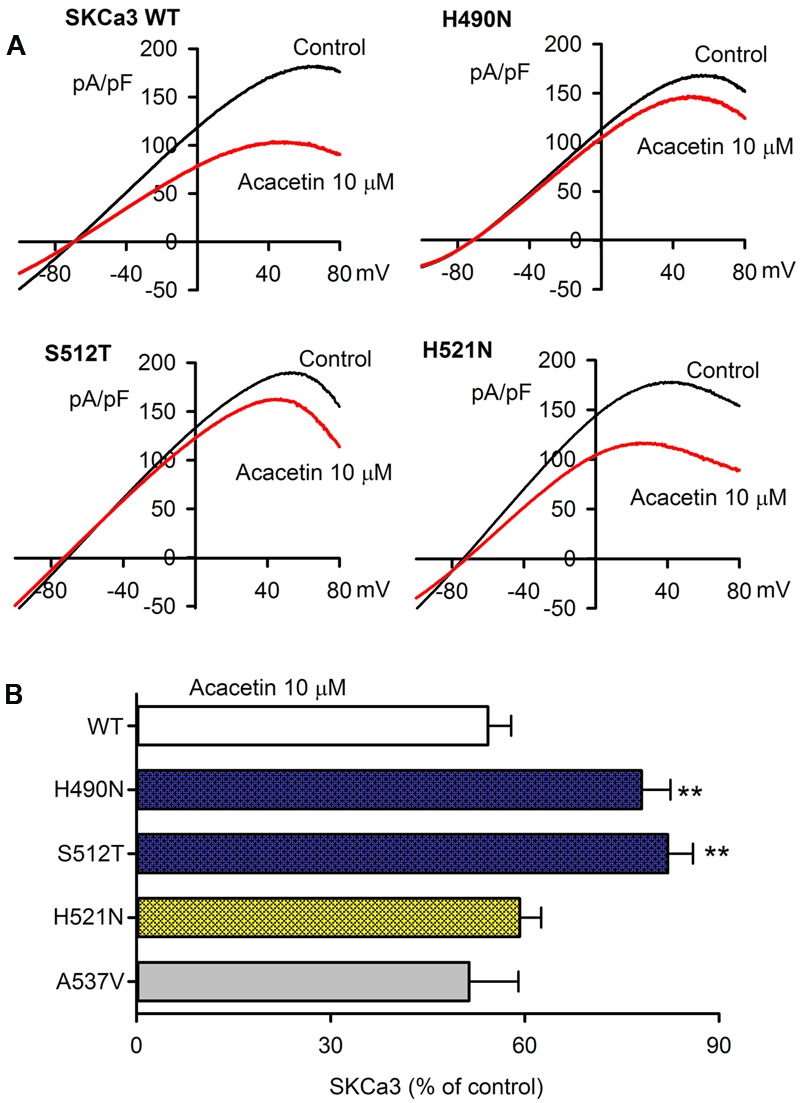
Effect of acacetin on WT and mutant SK_Ca_3 channel currents expressed in HEK 293 cells. **(A)** Current–voltage (*I–V*) relationships of SK_Ca_3 WT current, H490N, S512T, H521N were recorded in typical experiments with a 3-s voltage ramp from –100 to +80 mV in the absence and presence of 10 μM acacetin. **(B)** Percent values of 10 μM acacetin for inhibiting SK_Ca_3 WT current (*n* = 11, H490N current (*n* = 7, ^∗∗^*P* < 0.01 vs. WT), S512T current (*n* = 7, ^∗∗^*P* < 0.01 vs. WT), H521N current (*n* = 7, *P* > 0.05 vs. WT), or A537V current (*n* = 6) at +80 mV.

The potential molecular determinant of acacetin for inhibiting SK_Ca_ channels was investigated using SK_Ca_3 mutants, H490N, S512T, H521N, and A537V in P-loop helix and S6, generated by site-directed mutagenesis as described previously (Wu et al., 2013a,b). **Figure 4A** illustrates the *I–V* relationships of wild type (WT) SK_Ca_ current and mutant currents recorded in representative cells expressing WT SK_Ca_3 or the mutant H490N, S512T, or H521N with a voltage ramp protocol before (control) and after 10 μM acacetin. The inhibitory effect of acacetin for the mutant H490N and S512T currents was clearly reduced, compared with WT SK_Ca_3 current. **Figure 4B** illustrates the percent values of current inhibition by 10 μM acacetin for WT SK_Ca_3, and the mutants H490N, S512T, H521N, and A537V currents at +80 mV. Acacetin at 10 μM decreased the current by 45.7 ± 4.1% for WT SK_Ca_1 current (*n* = 11), 21.9 ± 4.5% for H490N current (*n* = 7, *P* < 0.01 vs. WT), 17.9 ± 3.9% for S512T current (*n* = 7, *P* < 0.01 vs. WT), 48.8 ± 3.5% for H521N current (*n* = 7, *P* > 0.05 vs. WT), and 40.8 ± 7.6% for A537V current (*n* = 6, *P* > 0.05 vs. WT), respectively.

**FIGURE 5:**
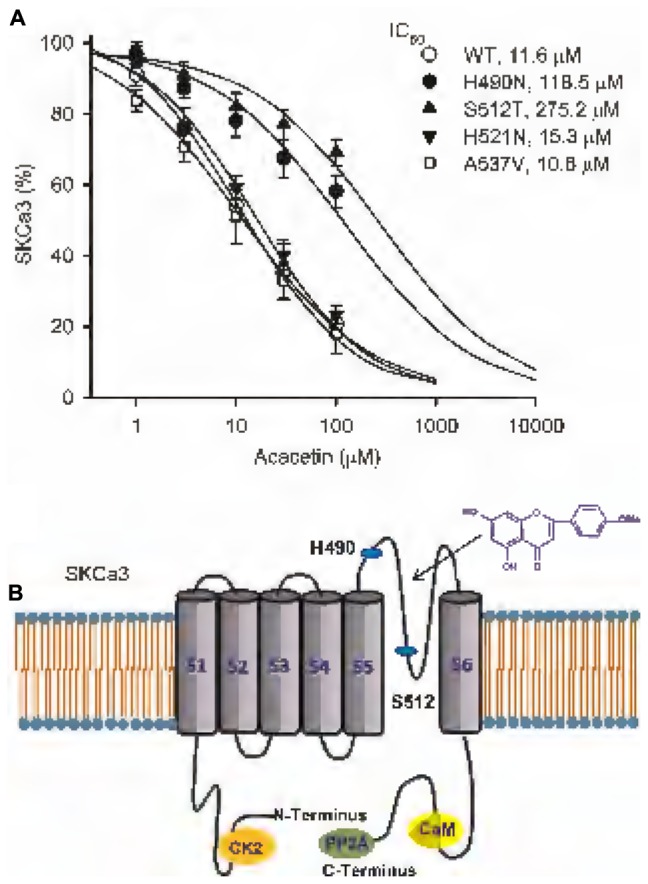
IC_50_ values of acacetin for inhibiting SK_Ca_3 WT, H490N, S512T, H521N, or 537V current in HEK 293 cells. **(A)** Concentration–response relationship curves of acacetin for inhibiting SK_Ca_3 WT current (+80 mV), H490N, S512T, H521N, or 537V (*n* = 6–9 for each concentration) was fitted to a Hill equation to obtain IC_50_ values of acacetin. **(B)** Schematic graph showing the putative binding sites of acacetin at H490, S512, and also H521 in the P-loop helix of human SK_Ca_3 channels.

**Figure 5A** displays the concentration–response relationships of acacetin for inhibiting WT SK_Ca_3 current, H490N current, S512T current, H521N current, and A537V current at +80 mV. The concentration-dependent inhibition curves were fitted to a Hill equation. The IC_50_ of acacetin was 11.6 μM for WT SK_Ca_3 current, 118.5 μM for H490N current, 275.2 μM for S512T current, 15.3 μM for H521N current, and 10.6 μM for A537V current, respectively. The efficacy of acacetin for inhibiting H490N current and S512T current was dramatically reduced, which suggests that acacetin blocks SK_Ca_3 channel by interacting with H490 and S512 in the P-loop helix of the channel (**Figure 5B**).

### Effect of Acacetin on IK_Ca_ Current

**FIGURE 6:**
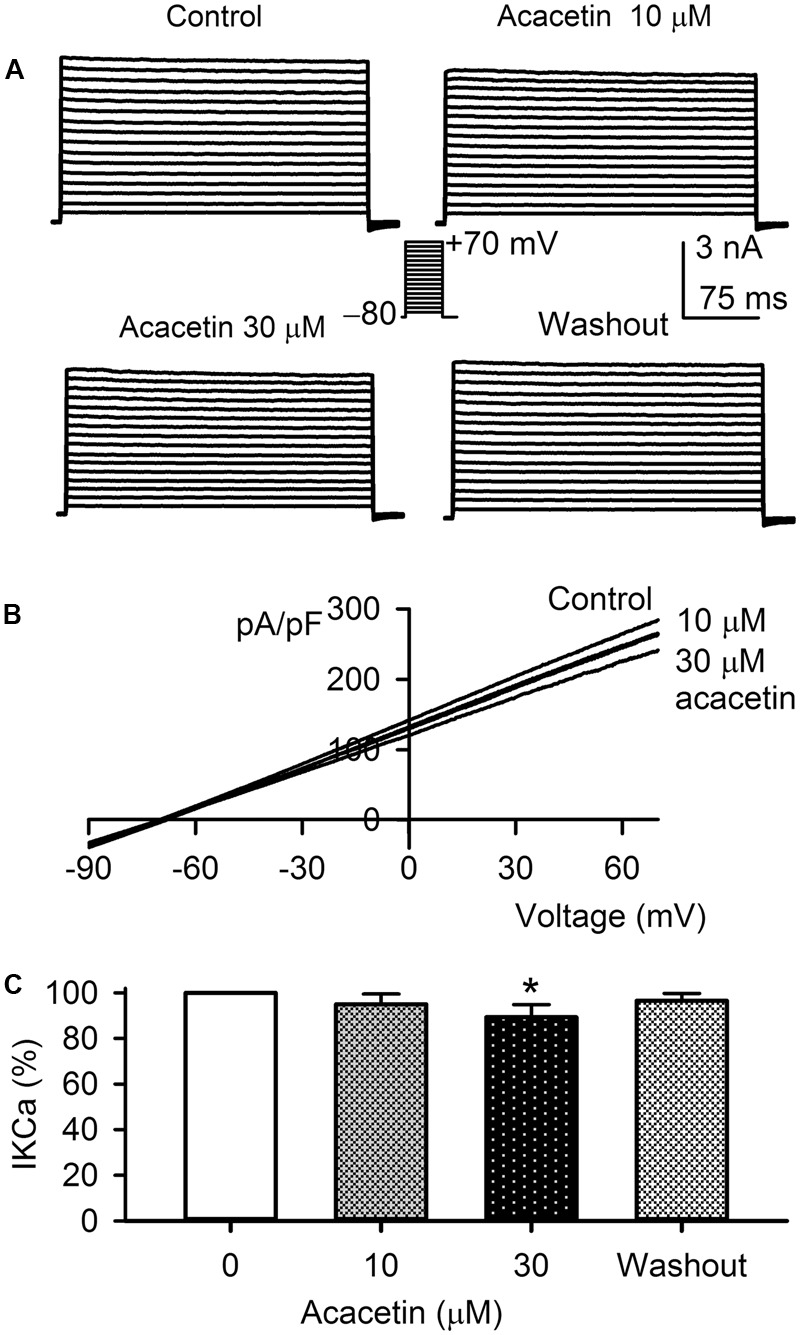
Effect of acacetin on IK_Ca_ channel stably expressed in HEK 293 cells. **(A)** IK_Ca_ current was activated in a representative cell expressing human *KCNN4* by 200-ms step voltages between –70 and +70 mV from a holding potential of –80 mV in the absence and presence of 10 or 30 μM acacetin. **(B)** Current–voltage (*I–V*) relationships of IK_Ca_ current were recorded in a typical experiment with a 3-s voltage ramp from –90 to +70 mV in the absence and presence of 10 and 30 μM acacetin. **(C)** Percent values of acacetin (10 or 30 μM) for inhibiting IK_Ca_ current (+70 mV, *n* = 7, ^∗^*P* < 0.05 vs. 0 μM acacetin).

The potential effect of acacetin on IK_Ca_ was determined in HEK 293 cell line expressing human *KCNN4*. The voltage-dependent IK_Ca_ current (**Figure 6A**) was recorded with the step voltages as shown in the inset in a typical experiment before and after application of acacetin. Acacetin (10 and 30 μM) slightly decreased the current, and the inhibition was partially recovered on washout. Similar results were observed for the *I–V* relationships of the current recorded with a voltage ramp in another representative cell (**Figure 6B**). IK_Ca_ shows a linear *I–V* relationship, similar to those previously recorded in HEK 293 cell line expressing IK_Ca_ (Girault et al., 2011). **Figure 6C** shows that acacetin (10 and 30 μM) decreased IK_Ca_ (+70 mV) to 95.0 ± 4.5% (*n* = 7, *P* > 0.05) and 89.3 ± 5.5% of control (*n* = 7, *P* < 0.05 vs. control, 0 μM), respectively. These results suggest that acacetin has a slight inhibition of IK_Ca_ current.

### Effect of Acacetin on BK_Ca_ Current

**FIGURE 7:**
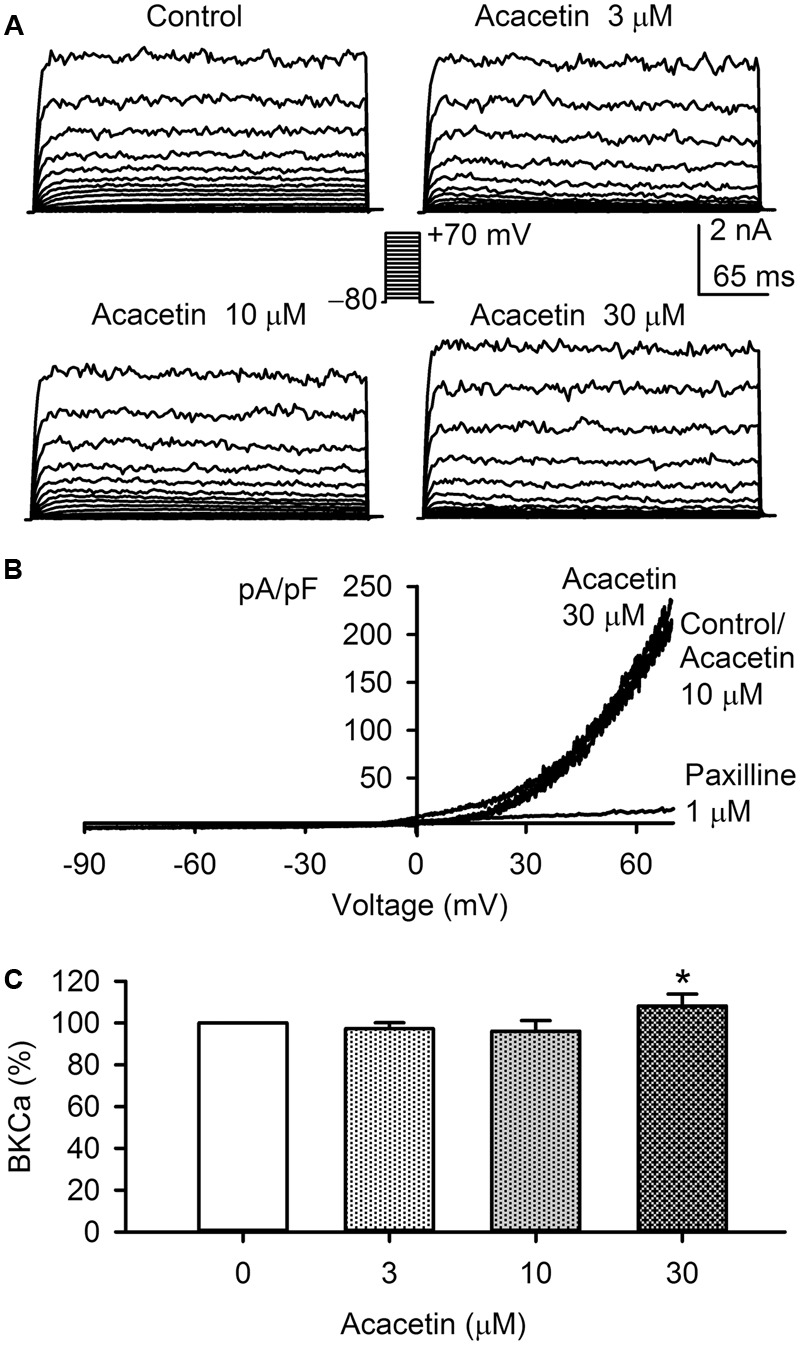
Effect of acacetin on BK_Ca_ channel stably expressed in HEK 293 cells. **(A)** BK_Ca_ current was activated in a representative cell expressing human *KCNMA1* by 200-ms step voltages between –70 and +70 mV from a holding potential of –80 mV in the absence and presence of 3, 10, or 30 μM acacetin. **(B)** Current–voltage (*I–V*) relationships of SK_Ca_3 current were recorded in a typical experiment with a 3-s voltage ramp from –90 to +70 mV in the absence and presence of 10 and 30 μM acacetin. **(C)** Percent values of acacetin (3, 10, or 30 μM) for increasing BK_Ca_ current (+70 mV, *n* = 7, ^∗^*P* < 0.05 vs. 0 μM acacetin).

The effect of acacetin on BK_Ca_ current was examined in HEK 293 cell line expressing human *KCNMA1* gene. Voltage-dependent BK_Ca_ current was recorded with the step voltage protocol as shown in the inset in a representative cell before and after application of acacetin (**Figure 7A**). Acacetin had no effect on BK_Ca_ at 3 and 10 μM, whereas it slightly increased the current at 30 μM. *I–V* relationships (**Figure 7B**) of BK_Ca_ current determined with a voltage ramp showed a similar response to acacetin. Acacetin did not affect the current at 10 μM, but slightly increased the outward component of BK_Ca_ current at 30 μM. The BK_Ca_ inhibitor paxilline (1 μM) almost fully suppressed the current. The percent values of BK_Ca_ current at +70 mV illustrated in **Figure 7C** show that no significant effect of acacetin was observed at 3 and 10 μM, whereas 30 μM acacetin increased the current to 108.1 ± 5.7% of control (*n* = 7, *P* < 0.05 vs. control). These results suggest that acacetin may stimulate BK_Ca_ channel at high concentration of 30 μM.

## Discussion

The present study provides the novel information that the natural flavone acacetin blocks the three SK_Ca_ channel subtypes: SK_Ca_1, SK_Ca_2, and SK_Ca_3, stably expressed in HEK 293 cells with similar efficacy. The IC_50_ values of acacetin for inhibiting SK_Ca_1, SK_Ca_2, and SK_Ca_3 are 12.4, 10.8, and 11.6 μM, respectively. Point mutagenesis of SK_Ca_3 channel reveals that acacetin mainly interacts with H490 and S512 in the P-loop helix of the channel. However, acacetin at a high concentration of 30 μM induces only a small decrease in IK_Ca_ channel and a small increase in BK_Ca_ channel stably expressed in HEK 293 cells. The very limited effect of acacetin on IK_Ca_ channel is similar to that reported previously for other SK_Ca_ channel blockers (Girault et al., 2011).

An earlier study demonstrated that SK_Ca_ channels were expressed in rat skeletal muscles, and sensitive to blocking by apamin (Blatz and Magleby, 1986). Then, the sequence of the transmembrane segments of SK_Ca_1, SK_Ca_2, and SK_Ca_3 are found 80–90% identical (Kohler et al., 1996). However, the three subunits have different sensitivity to blocking by apamin (SK_Ca_2 > SK_Ca_1 > SK_Ca_3), and are highly conserved among mammalian species, and are identified in many organisms from *Drosophila* to humans (Adelman et al., 2012). SK_Ca_ subunits assemble to form homomeric (Kohler et al., 1996) or heteromeric (Tuteja et al., 2010) tetramers. SK_Ca_ channels are identified in human and mouse atrial myocytes (Tuteja et al., 2005; Skibsbye et al., 2014), neurons (Church et al., 2015), and tumor cells (Girault et al., 2011, 2012; Gueguinou et al., 2014).

In the heart, activation of SK_Ca_ channel may be antiarrhythmic or proarrhythmic, depending on the myocardial pathophysiological conditions (Chang and Chen, 2015). Chua et al. (2011) reported that the SK_Ca_ channel current was heterogeneously upregulated in failing rabbit ventricles and SK_Ca_ blocker apamin suppressed post-shock shortening of action potential duration in the failing hearts with ventricular fibrillation. On the other hand, apamin induced ventricular arrhythmias in slowly paced failing rabbit ventricles (Chang et al., 2013). The proarrhythmic effect was also observed with apamin in isolated normal canine left atrium (Hsueh et al., 2013).

However, the results from other groups demonstrated that blockade of SK_Ca_ channels prolongs atrial effective refractory period, and SK_Ca_ channels are therefore considered as a promising therapeutic target in the treatment of AF (Diness et al., 2010, 2011; Qi et al., 2014; Haugaard et al., 2015). Several SK_Ca_ channel blockers, e.g., NS8593, UCL1684, *N*-(pyridin-2-yl)-4-(pyridin-2-yl)thiazol-2-amine (ICA) and apamin, have been used for anti-AF studies (Diness et al., 2010, 2011, 2015). In perfused guinea pig hearts, NS8593, UCL1684, and ICA effectively terminated AF induced with a combination of acetylcholine with electric stimulation (Diness et al., 2010). Injection of NS8593, UCL1684 or apamin reduced the duration of pacing-induced AF *in vivo* rat model (Skibsbye et al., 2011). UCL1684 and NS8593 had significant anti-AF effect in a rat paroxysmal AF with hypertension-induced atrial remodeling (Diness et al., 2011). Interestingly, in large animals such as dogs (Qi et al., 2014) and horses (Haugaard et al., 2015), intravenous administration of NS8593 terminated all induced AF episodes, increased atrial effective refractory period, and decreased AF duration and vulnerability without QTc interval prolongation, suggesting that SK_Ca_ channel blockers can be considered as promising anti-AF drugs. Moreover, recent studies showed that acute myocardial infarction might activate SK_Ca_ channels, and apamin, UCL-1684 or ICA reduced ventricular burden arrhythmia by prolonging ventricular action potential duration and effective refractory period in rats with acute myocardial infarction (Gui et al., 2013; Hundahl et al., 2017).

In this study, we demonstrated that acacetin inhibited SK_Ca_1, SK_Ca_2, and SK_Ca_3 channels in HEK 293 cell line expressing the corresponding genes. The blockade of SK_Ca_ channels by acacetin likely also contributes to the anti-AF effect observed in canine models in addition to blocking *I*_Kur_/Kv1.5, *I*_to_/Kv4.3, and *I*_K.ACh_ (Li et al., 2008; Wu et al., 2011, 2013a). These studies suggest that acacetin blocks multiple atrial-selective channels, and would be more effective in anti-AF than the blocker that specifically inhibits one type of atrial channel. However, whether acacetin, as apamin and other SK_Ca_ blockers, is effective in improving learning and memory (Adelman et al., 2012) remains to be studied in the future. Moreover, additional studies are required for clarifying whether the SK_Ca_ blocking effect of acacetin is related to its anti-cancer effect (Salimi et al., 2016; Zeng et al., 2017).

In our previous reports, we demonstrated that acacetin blocked Kv1.5 channel by binding to both its resting and open states by interacting with V505, I508, and V512 within the S6 domain (Wu et al., 2011), and inhibited the closed channel and blocked the open state of Kv4.3 by binding to both P-loop selectivity filter and S6 domain (Wu et al., 2013a). In the present study, we found that H490 and S512, but not H521, of P-loop helix are the binding sites of acacetin for blocking SK_Ca_3 channel. The pore blocking of SK_Ca_3 by acacetin is applicable to SK_Ca_1 and SK_Ca_2, because SK_Ca_1, SK_Ca_2, and SK_Ca_3 share the same sequence in the range of pore helix^1^. This differs from the molecular determinants of acacetin for blocking Kv1.5 or Kv4.3 channel. On the other hand, the acacetin blockade of SK_Ca_3 channel is different from the organic SK_Ca_ blocker NS8593 and the archetypical peptide SK_Ca_ blocker apamin. NS8593 interacts with S507 of P-loop helix and A532 of S6 domain (Jenkins et al., 2011), while apamin binds to a residue of S3–S4 extracellular loop of outside pore of the channel to produce a high-sensitivity block without selectivity filter contact (Weatherall et al., 2011). While the binding sites of various SK_Ca_ blockers differ, it is important to develop these potential blockers into feasible drug candidates for future clinical application. A water soluble prodrug of acacetin has been developed, which can be intravenously administered for future clinical application (Liu et al., 2016).

Acacetin showed increased BK_Ca_ current at concentration of 30 μM. Although the concentration for activating BK_Ca_ channel is greater than those of blocking *I*_Kur_/Kv1.5, *I*_K.ACh_, *I*_to_/Kv4.3, and also SK_Ca_ channels; this effect may account in part for the vascular dilation reported in a previous study (Calderone et al., 2004).

A limitation of the present study was that all the experiments were conducted only in HEK 293 line expressing SK_Ca_1, SK_Ca_2, or SK_Ca_3 channels and lack of data from native cardiomyocytes. However, this does not affect the conclusion that acacetin blocks SK_Ca_ channels. Future effort is required to obtain the data for the effect of acacetin on SK_Ca_ current in native cardiomyocytes from an animal species whose heart has no or less expression of *I*_Kur_/Kv1.5 and *I*_to_/Kv4.3, because acacetin also inhibits these currents in native human atrial myocytes (Li et al., 2008).

Collectively, the present study demonstrates for the first time that acacetin is a SK_Ca_ channel blocker and inhibits three subtypes of the SK_Ca_ channels stably expressed in HEK 293 cells. The SK_Ca_ channel blocking effect may be involved in its anti-AF property previously observed in experimentally induced AF in dogs.

## Author Contributions

K-HC, M-WJ, G-SX, YW, and G-RL conceived and designed the project. K-HC, HL, and H-YS conducted the experiments. K-HC, HL, H-YS, and G-RL analyzed the data. K-HC and G-RL prepared the manuscript. All authors read and approved the manuscript.

## Conflict of Interest Statement

The authors declare that the research was conducted in the absence of any commercial or financial relationships that could be construed as a potential conflict of interest.

## Footnotes

1 http://www.uniprot.org/uniprot

## Abbreviations

AF: atrial fibrillation
BK_Ca_: big/large conductance Ca^2+^-activated potassium channels
IK_Ca_: intermediate conductance Ca^2+^-activated potassium channels
K_Ca_: Ca^2+^-activated potassium channel
SK_Ca_: small conductance Ca^2+^-activated potassium channels
